# Blood pressure values and depression in hypertensive individuals at high cardiovascular risk

**DOI:** 10.1186/1471-2261-14-109

**Published:** 2014-08-26

**Authors:** Cilia Mejia-Lancheros, Ramón Estruch, Miguel Angel Martínez-González, Jordi Salas-Salvadó, Dolores Corella, Enrique Gómez-Gracia, Miquel Fiol, José Manuel Santos, Montse Fitó, Fernando Arós, Lluis Serra-Majem, Xavier Pintó, Josep Basora, José Vicente Sorlí, Miguel-Angel Muñoz, the PREDIMED Study Investigators

**Affiliations:** Department of Paediatrics, Obstetrics, Gynaecology and Preventive Medicine, Universitat Autònoma de Barcelona, Barcelona, Spain; Centro de Investigación Biomédica en Red de Fisiopatología de la Obesidad y Nutrición(CIBERobn), Instituto de Salud Carlos III, Madrid, Spain; The PREDIMED Study (Prevención con Dieta Mediterránea) Network (RD 06/0045), Instituto de Salud Carlos III, Madrid, Spain; The Department of Internal Medicine of Hospital Clinic, Institut d’Investigacions Biomèdiques August Pi I Sunyer, University of Barcelona, Barcelona, Spain; Preventive Medicine and Public Health, University of Navarra, Pamplona, Spain; Human Nutrition Department, Hospital Universitari Sant Joan, Institut d’Investigació Sanitaria Pere Virgili, Universitat Rovira i Virgili, Reus, Spain; The Department of Preventive Medicine, University of Valencia, Valencia, Spain; The Department of Preventive Medicine, University of Malaga, Malaga, Spain; Institute of Health Sciences (IUNICS), University of Balearic Islands, and Hospital Son Espases, Palma de Mallorca, Spain; The Department of Family Medicine, Primary Care Division of Seville, San Pablo Health Center, Seville, Spain; Cardiovascular Risk and Nutrition Research Group of Institut Mar d’Investigacions Mèdiques (IMIM)–Research Institute Hospital del Mar, Barcelona, Spain; The Department of Cardiology, University Hospital of Alava, Vitoria, Spain; The Department of Clinical Sciences, University of Las Palmas de Gran Canaria, Las Palmas, Spain; Lipids and Vascular Risk Unit, Internal Medicine, Hospital Universitario de Bellvitge, Hospitalet de Llobregat, Barcelona, Spain; Primary Care Division, Catalan Institute of Health, Institut d’Investigació en Atenció Primària Jordi Gol, Tarragona-Reus, Spain; Primary Care Division, Valencia Institute of Health, Valencia, Spain; Catalan Institute of Health, Institut d’Investigació en Atenció Primària Jordi Gol, Sardenya 375, Entlo, 08025 Barcelona, Spain

**Keywords:** Hypertension, Depression, Blood pressure

## Abstract

**Background:**

Hypertension and depression are both important risk factors for cardiovascular diseases. Nevertheless, the association of blood pressure on and depression has not been completely established. This study aims to analyze whether depression may influence the control of blood pressure in hypertensive individuals at high cardiovascular risk.

**Methods:**

Cross-sectional study, embedded within the PREDIMED clinical trial, of 5954 hypertensive patients with high cardiovascular risk factor profiles. The relationship between blood pressure control and depression was analyzed. A multivariate analysis (logistic and log-linear regression), adjusting for potential confounders (socio-demographic factors, body mass index, lifestyle, diabetes, dyslipidemia, and antihypertensive treatment), was performed.

**Results:**

Depressive patients, with and without antidepressant treatment, had better blood pressure control (OR: 1.28, CI 95%: 1.06-1.55, and OR: 1.30, CI 95%: 1.03-1.65, respectively) than non-depressive ones. Regarding blood pressure levels, systolic blood pressure values (mmHg) were found to be lower in both treated and untreated depressive patients (Log coefficient Beta: -1.59, 95% CI: -0.50 to -2.69 and Log coefficient Beta: -3.49, 95% CI: -2.10 to -4.87, respectively).

**Conclusions:**

Among hypertensive patients at high cardiovascular risk, the control of blood pressure was better in those diagnosed with depression.

**Trial registration:**

Unique identifier: ISRCTN35739639.

## Background

High blood pressure is a key risk factor for cardiovascular disease (CVD) incidence [1–3]. Its prevalence is globally estimated to be around 40%, and it accounted for approximately 7.5 million deaths in 2008 [1]. The latest health statistics from the United States of America have reported a hypertension prevalence of 33% among adults, and within this population only 53% reached target levels recommended by guidelines [4]. In addition to the classical risk factors, in the last decade the impact of psychosocial determinants, such as educational level and depression, has received increasing attention [5–7]. The prevalence of depression has risen dramatically in recent years; in fact, the World Health Organization (2012) reported more than 350 million people suffering from this condition worldwide [8]. Depression has been found to coexist with CVD and its associated risk factors such as hypertension, diabetes, overweight, and unhealthy life styles (smoking and harmful alcohol consumption) [7, 9, 10]. Evidence supporting the relationship between depression and blood pressure (BP) is however, complex and remains controversial [11–13]. In addition, evidence addressing the relationship between depression and hypertension control in hypertensive populations with respect to the control of hypertension are scarce. The optimal control of BP is an essential key to reduce the risk level of cardiovascular diseases [3]. Since depression is an additional cardiovascular risk factor as some antidepressant medication may modify BP levels. The present study was, therefore, aimed at determining the association of depression and BP control in elderly hypertensive people at high cardiovascular risk.

## Methods

### Study design and participants

Cross-sectional study using baseline data of hypertensive participants at high cardiovascular risk from the PREDIMED Study (Prevention with the Mediterranean diet). All details of the PREDIMED study including enrollment, design, population, methods, and main results have been described elsewhere [14, 15]. For the present work, all the hypertensive individuals (N = 5954) from the 7447 PREDIMED study participants were included. They fulfilled at least 1 of the 2 following criteria: 1) men (55–80 years old) and women (60–80 years old) with either type-2 diabetes or 2) three or more CVD risk factors (current smoking, dyslipidemia, body mass index (BMI) > =25 kg/m2, or family history of premature cardiovascular diseases). Exclusion criteria included previous history of CVD or other diseases such as food allergies, alcoholism, infection or acute inflammation, physical or mental disability, and those individuals taking part in any other clinical trial. Participants’ data were collected from medical records, clinical evaluation, and face to face interviews. Validated questionnaires were administered in order to obtain data on nutritional and physical activity habits [16–18]. Blood samples for laboratory tests were also obtained. Details on collection and measurements have been published elsewhere [13, 14].

### Ethical considerations

All participants signed an informed consent. The project was conducted in accordance with the Declaration of Helsinki and its subsequent amendments. The PREDIMED study was approved by the Institutional Review Board of Hospital Clinic (Barcelona, Spain), and registered in the Current Controlled Trials (number: ISRCTN3573963, http://www.controlled-trials.com/ISRCTN35739639).

### End points

#### Control of blood pressure

BP was considered well-controlled when systolic and diastolic blood pressure values (SBP, DBP) were below 140 mmHg and 90 mmHg, respectively, according to the recommendations of the European guidelines on cardiovascular disease prevention in clinical practice [18]. Both SBP and DBP were calculated based on the average of 4 measurements (two in the right arm and two in the left), taken in the primary care centers by well-trained primary care nurses. BP measures were assessed after a suitable resting period (more than 5 minutes) in a sitting position to avoid variability in the values due to patient movement/displacement. For the measurement of BP, a validated semiautomatic sphygmomanometer (Omron HEM-705CP) with an appropriately sized cuff for the arm of each participant was used. The determinations were performed at two minute intervals. The mean of the second and third measurement was recorded. When a difference > 5 mm Hg between the two determinations more than 5 mm Hg was detected the whole process was repeated.

### Main independent variable

#### Depression

Diagnosis of depression was established at the visit of inclusion in the study, by face to face interview, and the information was further confirmed in the clinical records. Participants were asked if some doctor had previously diagnosed them from depression. In Spain, the diagnosis of depression is carried out both by psychiatrists and family doctors. Usually, diagnostic is made following the American Psychological Association clinical criteria (DSM-IV) and those of the International Classification of Diseases (ICD) related to Mental and Behavioral Disorders or other mental health scales, included in the standardized health guidelines from the Spanish Ministry of Health. Antidepressant treatment was registered according to the patients’ self-reported information and consulting at the clinical records. In addition, participants were also asked whether they had taken any antidepressants in the previous month. They were finally classified as: *no diagnosis of depression* (no previous diagnosis of depression and not taking antidepressants), *untreated depression* (diagnosis of depression and not taking any antidepressants), and *treated depression* (diagnosis of depression and taking at least one of the following: selective serotonin reuptake inhibitors, non-selective monoamine reuptake inhibitors, monoamine oxidase A inhibitors, antidepressants in combination with psycholeptics, and other antidepressant agents). Participants were also asked about the time that had elapsed from since their first diagnosis of depression which was categorized as: ≤ 5 years, 6–10 years, and ≥ 11 years.

### Co-variables

The following co-variables were taken into consideration: age, sex, anxiolytic or sedative treatment, comorbidity (diabetes and dyslipidemia), and antihypertensive treatment (angiotensin-converting-enzyme inhibitor (ACE inhibitors), diuretics, calcium channel blockers, angiotensin II receptor antagonists, β-blockers, α-blockers, or other antihypertensive drugs).

### Potential confounding variables

Educational attainment, BMI, smoking habits, adherence to the Mediterranean diet pattern, physical activity, and alcohol intake were included in the analysis as they can be correlated with both depression and BP control.

### Statistical analysis

The descriptive analysis of categorical variables was expressed as percentages and quantitative variables by mean and standard deviation (SD). Bivariate analyses included chi square tests and ANOVA F-test. A multivariate logistic model was fitted to evaluate the association and estimate Odds Ratio (OR) between depression level and length, and good BP control of blood pressure. To confirm the association observed between well-controlled BP and depression, continuous variables were adjusted by log-linear regression for potential confounders (age, sex, educational attainment, anxyolitic or sedative treatment, BMI, lifestyle, hypertension co-morbidity, and antihypertensive treatment). Those statistically significant at bivariate analysis, or which could have any clinical relationship with the final end-points, were included in the multivariate models. An alpha level <0.05 and a confidence interval (CI) of 95% were employed for all statistical analyses. The goodness-of-fit logistic models were performed using Hosmer and Lemeshow test, and for linear model residual validation the Kolmogorov test was used.

## Results

Mean age of the participants was 67.2 years (SD 6.2), 60.5% were women, and 15.6% had depression. Amongst this group 71% had had depression diagnosed more than six years ago.

### Bivariate analysis

#### Characteristics of participants according to depression

Depressive participants were more commonly women, had low educational level, presented more obesity, and were sedentary and dyslipidemic. In contrast, members of this group were less frequently smokers and alcohol drinkers. With respect to BP, depressive participants had lower SBP and DBP values (Table 1). Participants with treated depression had a higher percentage of BP control, and a greater probability of receiving antihypertensive treatment. The percentage of patients receiving antidepressants was higher in those diagnosed more recently (less than 5 years).

**Table 1:** **Main characteristics of study population by depression and depression length**

	Depression levels				Time with depression diagnostic			
	No depression	Untreated depression	Treated depression ^a^		≤ 5 years	6-10 years	≥ 11 years	
	(N = 5027)	(N = 569)	(N = 358)		(N = 268)	(N = 159)	(N = 500)	
Characteristics of participants	%	%	%	P-value	%	%	P-value	P-value
**Age (years)** ^**†**^	67.3 (6.2)	66.9(6.0)	66.8(5.7)	0.137	66.4(6.0)	66.7(6.1)	67.1(5.8)	0.290
**Sex (Women)**	56.0	79.8	84.6	0.001	82.5	78.0	82.4	0.881
**Educational attainment**								
High level	7.6	4.9	6.4	0.003	7.1	7.5	4.0	0.280
Middle level	15.9	13.7	11.7		11.6	12.6	13.8	
Low level	76.6	81.4	81.8		81.3	79.9	82.2	
**Antidepressant treatment** ^**a**^	---	---	---	---	45.9	37.7	35.0	0.004
**Antianxiety or sedative treatment** ^**b**^	14.8	37.8	61.5)	0.001	45.5	48.4	47.2	0.700
**Body Mass Index( Kg/m** ^**2**^ **)** ^**†**^	30.1(3.8)	30.8(4.2)	30.5(3.6)	0.001	30.5(4.0)	30.8(3.8)	30.8(4.0)	0.521
**Life styles**								
Smoking^c^	38.3	24.6	23.5	0.001	22.8	24.5	24.8	0.543
Low adherence to the MeDiet pattern^d^	45.8	48.0	50.8	0.042	47.4	45.3	51.2	0.256
Sedentary^e^	35.0	44.6	50.0	0.001	47.8	45.3	46.6	0.800
High alcohol intake pattern^f^	21.7	16.2	10.3	0.001	14.6	15.1	13.2	0.567
**Hypertension comorbidity**								
Diabetes^g^	43.5	41.7	36.3	0.009	42.2	44.7	36.6	0.094
Dyslipidaemia^h^	73.7	76.4	83.2	0.001	81.0	73.6	79.8	0.894
**Blood pressure**								
Optimal control of blood pressure^i^	26.5	33.9	36.3	0.001	31.3	41.5	34.6	0.520
Systolic blood pressure( mmHg)^†^	151.1(19.0)	147.8(18.9)	150.4(18.9)	0.001	146.1(17.0)	145.8(18.7)	146.7(18.9)	0.832
Diastolic blood pressure( mmHg)^†^	83.7(10.2)	83.1(10.0)	82.6(9.4)	0.052	83.7(9.6)	83.3(10.4)	82.3(9.6)	0.143
Antihypertensive treatment^j^	80.6	85.8	87.2	0.001	85.5	85.5	86.8	0.680

^a^Taking at least one of the following drugs: Selective serotonin reuptake inhibitors, non-selective monoamine reuptake inhibitors, Monoamine oxidase A inhibitors, antidepressants in combination with psycholeptics, others antidepressant agents.

^b^Taking at least one of the following drugs: benzodiazepine derivatives, azaspirodecanedione derivatives, GABA (gamma-aminobutyric acid) analogues, natural antianxiety agents, ethanolamine derivatives, other anxiolytics, hypnotics and sedatives agents.

^c^Current smoker.

^d^Adherence to Mediterranean diet pattern < 9 points (median) on a scale of 0–14.

^e^Physical activity in leisure time < 1000 kcal/week in last year.

^f^Alcohol consumption more than 20gr. daily in men and 10 gr. daily in women.

^g^Diagnosis of diabetes.

^h^Diagnosis of dyslipidaemia.

^i^Systolic blood pressure <140 mmHg and diastolic blood pressure <90 mmHg.

^j^Taking at least one of the following antihypertensive drugs: angiotensin-converting-enzyme inhibitor (ACE inhibitors), diuretics, calcium channel blockers, angiotensin II receptor antagonists, Beta-blockers, α-blockers, or other antihypertensive drugs.

^†^Mean - Standard Deviation, p - value: ANOVA F test.

### Control of blood pressure

**Figure 1:**
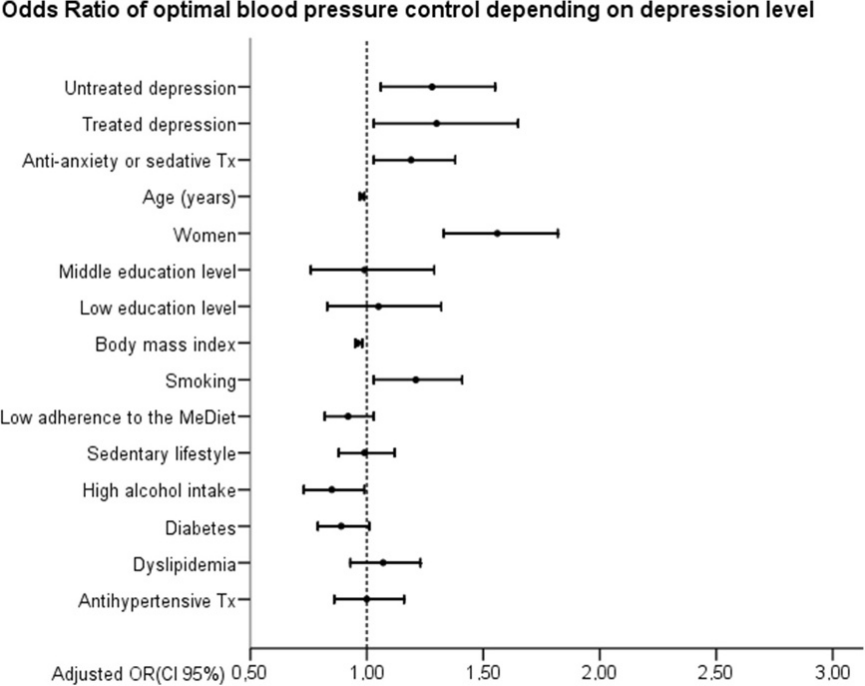
**Odds Ratios of optimal blood pressure control according to depression level.** Circles represent Odds Ratios, Horizontal lines indicate 95% confidence intervals and vertical line references OR = 1. Tx indicates treatment and MeDiet indicates the Mediterranean Diet. No depression is the reference category for the response variable.

After adjusting for the main co-variables (age, sex, anti-anxiety or sedative treatment, diabetes, dyslipidemia and anti-hypertensive treatment) and potential confounding factors (educational levels, BMI, smoking, diet pattern, and physical activity) depressive participants, with or without antidepressants, more frequently presented well-controlled BP than non-depressive ones (OR: 1.28, CI95%: 1.06-1.55 and OR: 1.30, CI95%: 1.03-1.65, respectively). Participants whose depression had been previously diagnosed between six and ten years had better BP control than the more recently diagnosed ones (OR: 1.62, CI95%: 1.07-2.45) (Figure 1). When considering BP as a continuous variable, only SBP figures were significantly lower in depressive patients, whilst DBP ones were unaffected (Table 2). Women, younger participants, and lower BMI were found to be related to better SBP and DBP levels.

**Table 2:** **Log-linear model coefficients of systolic and diastolic blood pressure according to depression level and adjusted co-variables**

	Natural logarithm of systolic blood pressure(mmHg) ^†^				Natural logarithm of diastolic blood pressure(mmHg) ^‡^			
	Coefficients ^§^	Lower CI 95% ^§^	Upper CI 95% ^§^	P-value	Coefficients ^§^	Lower CI 95% ^§^	Upper CI 95% ^§^	P-value
**Depression level**								
No Depression	Ref	Ref	Ref	Ref	Ref	Ref	Ref	Ref
Untreated depression^a^	-1.59	-2.69	-0.50	0.004	-0.59	-1.64	0.462	0.272
Treated depression^b^	-3.49	-4.87	-2.10	0.001	-0.85	-2.18	0.49	0.213
**Anti-Anxiety or sedative treatment** ^**c**^	-0.82	-1.66	0.02	0.056	-0.18	-0.99	0.62	0.657
**Age (years)**	0.25	0.20	0.31	0.001	-0.37	-0.42	0-.32	0.001
**Sex (Women)**	-2.60	-3.46	-1.75	0.001	-2.62	-3.44	-1.79	0.001
**Educational attainment**								
High level	Ref	Ref	Ref	Ref	Ref	Ref	Ref	Ref
Middle level	0.96	-0.45	2.37	0.183	1.09	-0.27	2.44	0.117
Low level	0.13	-1.12	1.38	0.841	-0.42	-1.62	0.78	0.491
**Body Mass Index( Kg/m** ^**2**^ **)**	0.24	0.16	0.33	0.001	0.42	0.34	0.50	0.001
**Smoking** ^**d**^	-0.80	-1.65	0.05	0.064	-0.76	-1.57	0.05	0.067
**Low adherence to the MeDiet pattern** ^**e**^	0.11	-0.56	0.78	0.746	-0.01	-0.65	0.64	0.989
**Sedentary** ^**f**^	0.17	-0.46	0.80	0.600	-0.07	-0.68	0.54	0.816
**High alcohol intake** ^**g**^	0.64	-0.17	1.45	0.122	0.72	-0.06	1.50	0.069
**Diabetes** ^**h**^	1.28	0.62	1.94	0.001	-1.94	-2.57	-1.30	0.001
**Dyslipidemia** ^**i**^	-0.34	-0.34	0.41	0.370	-0.60	-1.31	0.12	0.104
**Antihypertensive treatment** ^**j**^	0.55	-0.26	1.37	0.182	0.46	-0.32	1.24	0.252

^a^Diagnosis of depression and not taking any antidepressant drugs.

^b^Diagnosis of depression and taking at least one of the following drugs: Selective serotonin reuptake inhibitors, non-selective monoamine reuptake inhibitors, Monoamine oxidase A inhibitors, antidepressants in combination with psycholeptics, others antidepressant agents.

^c^Taking at least one of the following drugs: benzodiazepine derivatives, azaspirodecanedione derivatives, GABA (gamma-aminobutyric acid) analogues, natural antianxiety agents, ethanolamine derivatives, other anxiolytics, hypnotics and sedatives agents.

^d^Current smoker.

^e^Adherence to Mediterranean diet pattern < 9 points (median) on a scale of 0–14.

^f^Physical activity in leisure time < 1000 kcal/week in last year.

^g^Alcohol consumption more than 20gr. daily in men and 10 gr. daily in women.

^h^Diagnosis of diabetes.

^i^Diagnosis of dyslipidaemia.

^j^Taking at least one of the following antihypertensive drugs: angiotensin-converting-enzyme inhibitor (ACE inhibitors), diuretics, calcium channel blockers, angiotensin II receptor antagonists, Beta-blockers, α-blockers, or other antihypertensive drugs.

^†^Kolmogorov-Smirnov Test (p value): 0.271.

^‡^Kolmogorov-Smirnov Test (p value): 0.56.

^§^Coefficients values multiplied by 100.

## Discussion

In the present study we found that depressive, hypertensive participants at high cardiovascular risk had better BP values.

Although depression is considered an independent risk factor for hypertension incidence, and a number of authors have found it related to higher BP levels [19–21], its role in the control of BP values remains unclear [22]. Limited data have reported that hypertensive patients taking antidepressants have lower blood pressure levels [23]. One possible explanation for the effect of antidepressants on lowering blood pressure could be a reduction in vagal activity, decreased heart rate variability and baroreflex sensitivity [24], and neuro-endocrine pathways [25–29]. Our results concur with other studies performed in general populations [12, 13, 24, 30]. Research analyzing a group of people with hypertension who were taking antihypertensive drugs has also shown that individuals with episodes, or symptoms of depression, tended to have lower SBP and DBP [31]. It is not clear whether depression is the cause or the consequence of differences in the control of BP values [32, 33]. Confounders related to both hypertension and depression, such as physical activity, low-fat diet, non-smoking, and alcohol intake, were included in our analysis [34]. Some antidepressant, anti-anxiety, and antipsychotic agents, either alone or in combination with cardiovascular therapies including antihypertensive drugs, have been reported to induce a drop in BP [35–37]. Our participants diagnosed with depression, and those taking antidepressant treatments, received more antihypertensive drugs. Nevertheless, the association observed between depression and better blood pressure values persisted after adjusting for this variable in the multivariate analysis, which indicates that this association may be independent, as has been shown in previous studies [24, 33].

It could be hypothesized, moreover, that the frequent use of health services by depressive patients could contribute to an accurate follow-up and good control of their hypertension. The Spanish Health System guarantees a free and universal access to primary healthcare services. In addition, family doctors have access to well-established chronic care protocols, which ensure the better control and follow-up of patients with co-morbidity (hypertension and depression).

### Implication of our results

Our findings indicate the relevance of performing a holistic approach to the co-morbidity when tackling the care of chronic patients attended in primary care. Preventions among family physicians toward the use of antidepressants in hypertensive patients with depression should be addressed individually since many studies have shown an improvement in BP control.

### Study limitations and strengths

The cross-sectional design of our study does not allow causal inferences to be drawn. Future observational research studies are needed to establish the role of psychosocial factors in the good control of cardiovascular risk factors and the prognosis of cardiovascular diseases, especially in hypertensive individuals or those at high cardiovascular risk.

For reasons of statistical power the different antidepressants were grouped together. It is possible that a larger sample could establish variations according to the antidepressant analyzed. The time elapsed from the first diagnosis of depression could not be used as a proxy for the current prevalence of depression as the only way to establish the current state of the disease is through the prescription of antidepressants, and no specific tests were conducted in the participants. We had information about the family history of cardiovascular diseases history but none concerning about family history of depression and hypertension.

## Conclusion

Among hypertensive patients at high cardiovascular risk, blood pressure was better controlled in those diagnosed with depression.

## Authors’ information

This work has been carried out within the framework of the Predimed Study, one of the largest clinical trials ever performed to date regarding the association of a Mediterranean Intervention on cardiovascular morbi-mortality. The network consisted of more than seventeen different multidisciplinary research groups including experts on nutrition and internal medicine, cardiologists, and family physicians.

The main outcomes of the study were published last year [14].

## Abbreviations

CVD: Cardiovascular diseases
BP: Blood pressure
BMI: Body mass index
SBP: Diastolic blood pressure
DBP: Diastolic blood pressure
SD: Standard deviation
OR: Odds ratio
CI: Confidence interval.
